# On the Effects of Artificial Feeding on Bee Colony Dynamics: A Mathematical Model

**DOI:** 10.1371/journal.pone.0167054

**Published:** 2016-11-22

**Authors:** Juliana Pereira Lisboa Mohallem Paiva, Henrique Mohallem Paiva, Elisa Esposito, Michelle Manfrini Morais

**Affiliations:** 1 UNIFESP - Universidade Federal de Sao Paulo (*Federal University of Sao Paulo*), Rua Talim, 330, Sao Jose dos Campos, Brazil, 12231-280; 2 ITA - Instituto Tecnologico de Aeronautica (*Aeronautical Institute of Technology)*, Praca Marechal Eduardo Gomes, 50, Sao Jose dos Campos, Brazil, 12228-900; 3 UNIFESP - Universidade Federal de Sao Paulo, Rua Conceicao, 329, Diadema, Brazil, 09920-000; Universidade de Sao Paulo Faculdade de Filosofia Ciencias e Letras de Ribeirao Preto, BRAZIL

## Abstract

This paper proposes a new mathematical model to evaluate the effects of artificial feeding on bee colony population dynamics. The proposed model is based on a classical framework and contains differential equations that describe the changes in the number of hive bees, forager bees, and brood cells, as a function of amounts of natural and artificial food. The model includes the following elements to characterize the artificial feeding scenario: a function to model the preference of the bees for natural food over artificial food; parameters to quantify the quality and palatability of artificial diets; a function to account for the efficiency of the foragers in gathering food under different environmental conditions; and a function to represent different approaches used by the beekeeper to feed the hive with artificial food. Simulated results are presented to illustrate the main characteristics of the model and its behavior under different scenarios. The model results are validated with experimental data from the literature involving four different artificial diets. A good match between simulated and experimental results was achieved.

## Introduction

Beekeeping is an activity dependent on environmental resources. Bees collect nectar from flowers and take it to honeycombs, where it is converted to honey, which is the energetic food resource. Similarly, the forager bees collect pollen from flowers and store it in the honeycomb, where it is fermented and becomes bee bread, which provides protein, lipids, minerals and vitamins. Bees depend on floral resources and the collected pollen is used to feed the workers, especially in the early stages of adult life (nurses), responsible for producing food secretions for the queen and brood. The workers require large amounts of protein for activation of glands in the head and thorax, responsible for producing these foods [1, 2].

During some periods of the year (such as winter or the dry season), food resources are drastically reduced. With little natural food available, there is a reduction in the number of worker bees in the hive. This food decline also causes reduction of the queen’s laying, diminishes the survival rate of individuals, renders the colony susceptible to disease, and increases rates of absconding or abandonment [3, 4]. Moreover, in times of dearth, pollen reserves in the combs and protein reserves in the bees are soon spent. Therefore, supplementation with pollen or pollen substitute is required to maintain strong colonies for pollination and honey production [5].

To mitigate the effects of dearth periods, beekeepers can use artificial diets to supplement colony needs. These supplements must have balanced formulas that meet the nutritional needs of the bees, ensuring the survival and good performance of the colony. However, it should be noted that artificial food resources are not able to efficiently replace natural food (bee bread) because this natural protein source is more palatable and nutritionally complete, meeting all the requirements of the bees [2, 4, 6–8].

When the colonies are fed with artificial diets during these critical periods, there is an improvement in the physiological response of the animals measured through parameters such as hive weight gain, reproductive performance, disease resistance, increased number of individuals in the hive, and longevity. A well-nourished hive can survive critical periods, maintaining the queen’s egg laying and generating healthy offspring. The colony is then prepared to collect more pollen when it becomes available again, renewing food stocks and allowing for honey production [9, 10].

Considering the importance of finding adequate food sources for bees, laboratory and field tests have been developed in order to test ingredients and formulas that have the potential to meet the nutritional needs of these insects. Many different ingredients and formulations have been tested under various climatic and environmental conditions. Furthermore, mathematical models have been developed in order to predict colony population dynamics. These mathematical tools can describe the laboratory or field dynamics and are, therefore, a useful resource.

The problem of simulating bee population dynamics has been the subject of various studies. Experimental data has been used as a basis to develop complex models [11–14] that can account for the specific conditions the studies delineated.

Simpler models have also been proposed, involving differential equations and abstract concepts. In spite of their relative mathematical simplicity, these models are more appropriate to analyze general factors that may affect bee population dynamics and lend themselves better to hypothesis testing [15]. This is the case of the framework proposed by Khoury *et al.* [16, 17], which describes a colony population dynamics and has been used as a basis for studies by Russel *et al.* [18], Betti *et al.* [19], and Perry *et al.* [20].

Similarly, Sumpter and Martin [21] and Eberl *et al.* [22] used a model with three differential equations to describe the effects of a virus in the dynamics of colonies infested by the Varroa mite. Dennis and Kemp [15] proposed a model that is governed by a single differential equation; it was used to perform an extensive analysis of Allee effects on the colony, as well as an analysis of ecological resilience.

A detailed review of mathematical models to describe bee population dynamics was presented by Becher *et al.* [23], who analyzed 31 models, classified as colony, varroa, and foraging models.

However, no model has analyzed the effects of artificial feeding on bee population dynamics. This paper proposes a new model with this intent.

Perry *et al.* [20] have briefly considered the effect of supplemental feeding on the hive population dynamics. Their analysis has been performed considering that the beekeeper introduces extra natural food in the hive, i.e., that the food gathered by the bees and the extra food provided by the beekeeper are of the same nature. Our approach is different, involving a more detailed model describing scenarios in which bees are fed with artificial diets.

In the following sections, a detailed description of the proposed model is presented, followed by simulation results, validation with experimental data, and concluding remarks.

## The Proposed Model

The model proposed in this paper is based on the framework established by Khoury *et al.* [16, 17]. In the classification proposed by Becher *et al.* [23], this model can be considered a colony model.

As in most previous works, the current model takes into consideration only female bees (workers), because drones have little influence on colony dynamics. Only a small part of the colony is composed of drones, and their only function in the colony is breeding. Therefore, their influence can be neglected.

As in Khoury *et al.* [17], the model proposed here includes both young bees, which work in the hive, and older bees, which are foragers. Their numbers are indicated respectively by *H* (hive bees) and *F* (forager bees). Bees in the colony are recruited to become foragers following a recruitment function *R*(⋅). Stored natural food, including both nectar and pollen, is indicated by *f*, in grams. The number of uncapped brood cells is indicated by *B* and includes eggs and larvae but not pupae. Time is expressed in days.

A new variable is proposed here to account for artificial food availability. Artificial food, in grams, is denoted by *f*_*a*_.

The rate of change in brood cells follows a differential equation composed by two terms: the number of eggs that survive to become brood and the number of brood cells that produce new hive bees. This is represented by the following equation [17]:

$$\begin{array}{c} \frac{dB}{dt}=LS\left(\cdot \right)-\phi B \end{array}$$ (1)

where *L* is the number of eggs laid daily by the queen, *S*(⋅) is the survival function and *ϕ*, 0 ≤ *ϕ* ≤ 1, is the fraction of brood that ecloses as bees per day.

The survival function presented in [17] is a function of stored natural food *f* and number of hive bees *H*. This function is extended here to include the amount of stored artificial food *f*_*a*_.

Initially, we define the function *T*(*f*) as:

$$\begin{array}{c} T\left(f\right)=\frac{d^{2}}{f^{2}+d^{2}} \end{array}$$ (2)

*T*(*f*) is a sigmoid function that will converge to 0 if *f* ≫ *d* and to 1 if *f* ≪ *d*, where *d* is a parameter to be chosen. *T*(*f*) is used to weight the effect of stored artificial food in the colony dynamics. It is multiplied by *f*_*a*_, meaning that, if there is enough natural food available, *T*(*f*)*f*_*a*_ will approach zero and the effects of *f*_*a*_ will be negligible. Therefore, *T*(*f*) can be regarded as the likelihood that bees consume stored artificial food as a function of stored natural food.

We introduced a parameter *β* > 0 associated with the quality of the artificial food. It means that one gram of artificial food replaces *β* grams of natural food. The higher the value of *β*, the better the quality of the artificial diet. A value of *β* = 1 would mean artificial food as good as the natural resource. Usually, *β* < 1 because artificial food is not expected to be as nutritious as the natural alternative. It should be noted that this is a simplification: the quality of natural and artificial food is compared in terms of amount of food consumed, instead of nutritional balance. A comparison in terms of nutritional balance would require a much more complex approach that is beyond the scope of the present work.

The survival function is described by:

$$\begin{array}{c} S\left(H,f,f_{a}\right)=\left(\frac{H}{H+\nu }\right)\left(\frac{{\left[f+\beta T\left(f\right)f_{a}\right]}^{2}}{{\left[f+\beta T\left(f\right)f_{a}\right]}^{2}+b^{2}}\right) \end{array}$$ (3)

which is composed of two multiplicative terms. The first is the same proposed in [17] and represents the influence of the number of hive bees over *S*(⋅). In this term, *ν* is a parameter that defines how fast it will converge to 1 as the number of hive bees *H* grows. The second multiplicative term in Eq (3) represents the effect of artificial and natural food stores on *S*(⋅). As in the first term, *b* is a parameter that defines how fast the second term will converge to 1 as the amount of artificial and natural food stores grow. [*f*+*βT*(*f*)*f*_*a*_] represents the amount of stored food deemed palatable by the bees, and adjusted for based on the quality of the artificial food.

The difference between the formula presented in Eq (3) and the original expression in [17] is the presence of the term *βT*(*f*)*f*_*a*_. This additional term ensures that the availability of stored artificial food increases the egg survival rate.

The rate of change in the number of hive bees follows a differential equation that is composed of two terms: the number of brood cells that develop into adult bees and the number of bees recruited to become foragers. This is represented by the following equation [17]:

$$\begin{array}{c} \frac{dH}{dt}=\phi B\left(t-\tau \right)-HR\left(\cdot \right) \end{array}$$ (4)

where the parameter *τ*, in days, indicates the delay required for brood to emerge from pupation. *R*(⋅) is a recruitment function, representing the fraction of hive bees that become foragers. Since the hive is much safer than the external environment, the death rate of hive bees is considered negligible and not included in the model.

In the formula presented in this paper, *R*(⋅) is also extended to account for the provision of artificial food. The recruitment function is defined as:

$$\begin{array}{c} R\left(H,F,f,f_{a}\right)=\alpha_{min}+\alpha_{max}\left(\frac{b^{2}}{({\left[f+\beta T\left(f\right)f_{a}\right]}^{2}+b^{2}}\right)-\sigma \frac{F}{F+H} \end{array}$$ (5)

which is based on the formula from [17] but adds the term *βT*(*f*)*f*_*a*_, which accounts for how the availability of stored artificial food reduces the recruitment rate of hive bees. The parameter *α*_*min*_ represents the recruitment rate when there is no shortage of food. *α*_*max*_ is the additional rate when the amount of food is low, in which case, more hive bees will be recruited to become foragers. *σ* is a parameter associated to social inhibition, that is, how the presence of foragers slows the rate of hive bees becoming foragers.

The rate of change in the number of foragers is the difference between the number of hive bees that are recruited and their mortality. This difference is expressed in the following equation [17]:

$$\begin{array}{c} \frac{dF}{dt}=HR\left(\cdot \right)-mF \end{array}$$ (6)

where *m*, 0 ≤ *m* ≤ 1, is a constant describing the rate of foragers dying daily. Its inverse, 1/*m*, represents the average flight span of a forager, in days. An alternative mortality expression has been proposed in [15], where bee mortality is described using a function that is higher for a small number of foragers and converges to a constant rate only if the number of foragers is high enough. However, for the sake of simplicity, we have chosen to maintain a constant rate of forager death.

The equation describing the daily rate of change in stored natural food is given by the difference in the amount of food brought to the colony by the foragers and the food consumed by adult bees and larvae in the colony.

In the original approach of [17], the amount of food brought to the colony daily by each forager was a constant value. Here, we have added an efficiency function *μ*(⋅), 0 ≤ *μ*(⋅)≤1, which accounts for variations in the availability of natural food, considering environmental factors such as a shortage of food in winter. In our model, function *μ*(⋅) is particularly important to allow for analysis of the dynamics when there is a shortage of natural food. A similar approach was adopted by Russel *et al.* [18], who included a seasonal coefficient that decreases the daily amount of food collected by each forager. This coefficient is based on experimental work by Harbo [24] and assumes a fixed value for each month of the year. We have opted for a more general approach, with a function *μ*(⋅) that can assume several forms. For instance, it can represent the shortage of food during the dearth season in some regions of South America, where the dry seasons do not coincide with winter.

In [17], where all consumed food is of natural origin, the daily food consumption in the hive Γ is quantified as:

$$\begin{array}{c} \Gamma =\gamma_{A}\left(F+H\right)+\gamma_{B}B \end{array}$$ (7)

where *γ*_*A*_ is the average amount of natural food consumed daily by each hive bee and by each forager and *γ*_*B*_ is the average amount of natural food consumed daily by each uncapped brood item.

We adopt the same expression in Eq (7) to represent the total daily food consumption in the hive. However, since part of the consumed food may be artificial, we define a new function *U*(⋅), 0 ≤ *U*(⋅)≤1, to represent the portion of the total food needed daily by the colony that is obtained by natural sources. Hence, in our model, the total food consumption Γ expressed in Eq (7) is multiplied by *U*(⋅) to represent the natural food consumption. The remaining food required by the colony will be obtained from synthetic food sources.

Therefore, the equation that describes the rate of variation of stored natural food is given by:

$$\begin{array}{c} \frac{df}{dt}=\mu \left(\cdot \right)cF-\left(\gamma_{A}\left(F+H\right)+\gamma_{B}B\right)U\left(\cdot \right) \end{array}$$ (8)

where *c* is the maximum food brought in daily to the colony by each forager.

We assume that the fraction of natural food *U*(⋅) can be estimated by the availability of natural food in the hive as a ratio of total food available in the hive, where total available food is adjusted for bee preference for natural food. Therefore, the following equation is used to estimate the value of *U*(⋅):

$$\begin{array}{c} U\left(f,f_{a}\right)=\{\begin{array}{cc} \frac{f}{f+T\left(f\right)f_{a}} & ,[f+T\left(f\right)f_{a}]\neq 0\\ 0 & ,[f+T\left(f\right)f_{a}]=0 \end{array} \end{array}$$ (9)

As mentioned before, if there is enough natural food in storage, *T*(*f*) will be 0. In this case, according to Eq (9), *U*(⋅) will be 1. Thus, artificial food is consumed only if there is a shortage of natural food.

Finally, the equation describing the daily rate of change in artificial food is given by the difference between the amount of food provided to the colony by the beekeeper and the artificial food consumed by each bee and brood item in the colony. It is described by:

$$\begin{array}{c} \frac{df_{a}}{dt}=z\left(\cdot \right)-\left(\frac{\gamma_{A}}{\beta }\left(F+H\right)+\frac{\gamma_{B}}{\beta }B\right)\left(1-U\left(\cdot \right)\right) \end{array}$$ (10)

where (1 − *U*(⋅)) complements the factor of *U*(⋅) used in Eq (8). *γ*_*A*_ and *γ*_*B*_ are the same values defined in Eq (8); they are divided by *β* in order to represent that each gram of natural food not consumed in Eq (8) is replaced by 1/*β* grams of artificial food. *z*(⋅) is a function indicating the rate at which the beekeeper provides artificial food to the colony. Several different functions *z*(⋅) can be adopted to simulate different approaches; for instance, a constant value of *z*(⋅) would indicate a constant amount of food introduced daily by the beekeeper into the colony.

**Fig 1:**
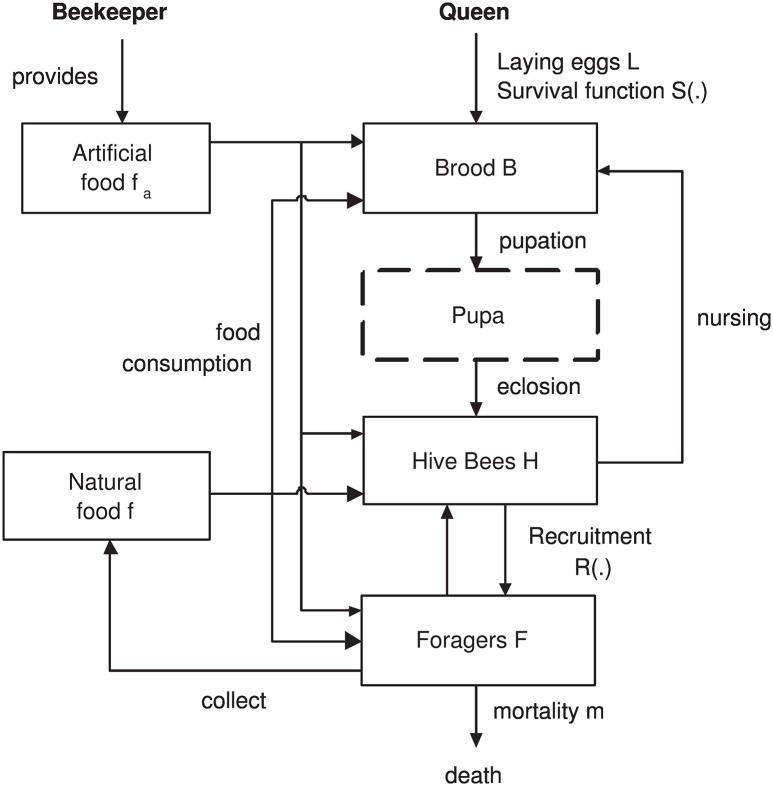
Model overview (extended from [17]).

Fig 1 presents an overview of the proposed model, summarizing the interactions previously described in this section. As previously discussed, it is an extension of the model presented in [17]. As in [17], the box indicating “Pupa” is represented with dashed borders because pupae are not directly represented in the model, being simulated as the time delay *τ* between the brood entering pupation and their emerging as adults.

## Results and Discussion

### Functions *T*(*f*), *U*(*f*, *f*_*a*_), and *S*(*H*, *f*, *f*_*a*_)

**Fig 2:**
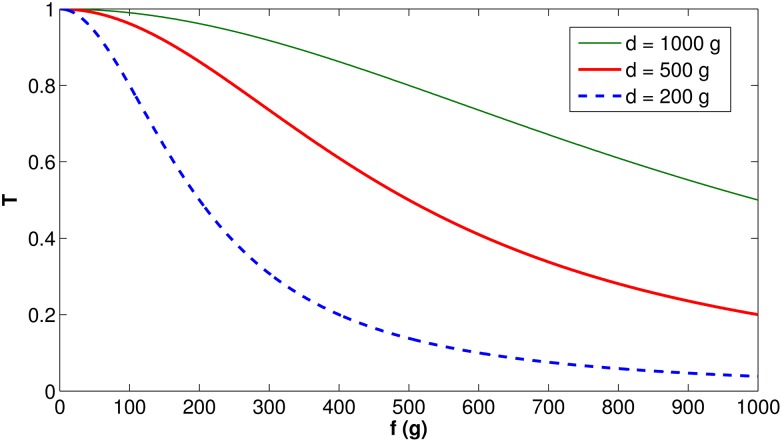
Function *T*(*f*) behavior, for different values of *d*.

Fig 2 depicts the behavior of the *T*(*f*) function (defined in Eq (2)), illustrating how fast *T*(*f*) converges to 0 for different values of *d*. *d* is a parameter associated with the palatability of the artificial food. Less palatable food will present lower values of *d*, which implies faster convergence of *T*(*f*) to 0, meaning more rejection by the bees than more palatable food.

In the subsequent analysis, *d* = 500 g will be adopted for illustrative purposes.

**Fig 3:**
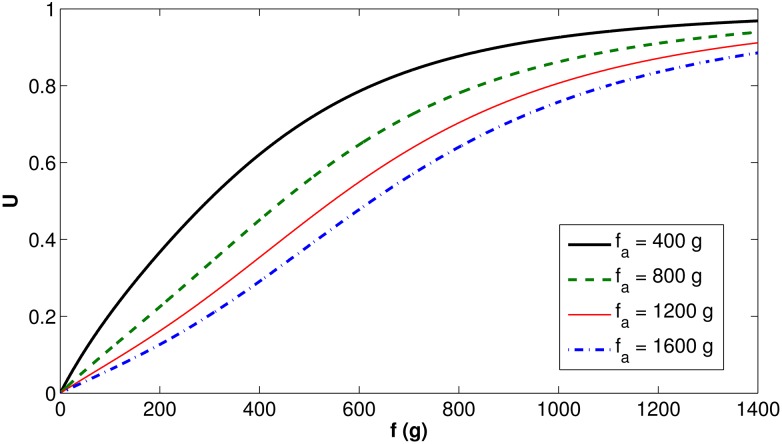
Function *U*(*f*, *f*_*a*_) behavior as a function of *f*, for fixed values of *f*_*a*_.

**Fig 4:**
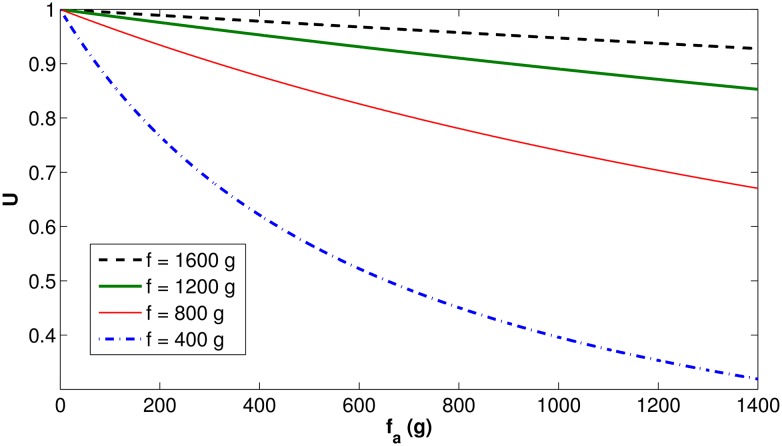
Function *U*(*f*, *f*_*a*_) behavior as a function of *f*_*a*_, for fixed values of *f*.

Figs 3 and 4 present the behavior of the *U*(*f*, *f*_*a*_) function (defined in Eq (9)), first for fixed values of *f*_*a*_, then for fixed values of *f*. As previously explained, *U*(*f*, *f*_*a*_) represents the fraction of natural food consumed as a function of the total food needed.

Fig 3 shows that, when the amount of natural food increases, the fraction of natural food *U*(*f*, *f*_*a*_) also increases, converging from 0 at *f* = 0 g to 1 as *f* increases. The speed of convergence depends on how much artificial food is stored in the hive, but, in any case, *U*(*f*, *f*_*a*_) will quickly approach 1.

Fig 4 shows that, when the amount of artificial food increases, the fraction of natural food *U*(*f*, *f*_*a*_) decreases, starting at 1 and slowly decreasing to 0. It is worth noting the slow decrease to zero, especially if the amount of available natural food is high. It means that, in the presence of sufficient natural food, only small amounts of artificial food will be consumed. This is a representation of the natural behavior observed in experimental tests, in which bees prefer natural food, as long as it is available [25]. This behavior clarifies the importance of the function *T*(*f*): without this function, the model would not be able to emulate the preference of bees for natural food over artificial food.

**Fig 5:**
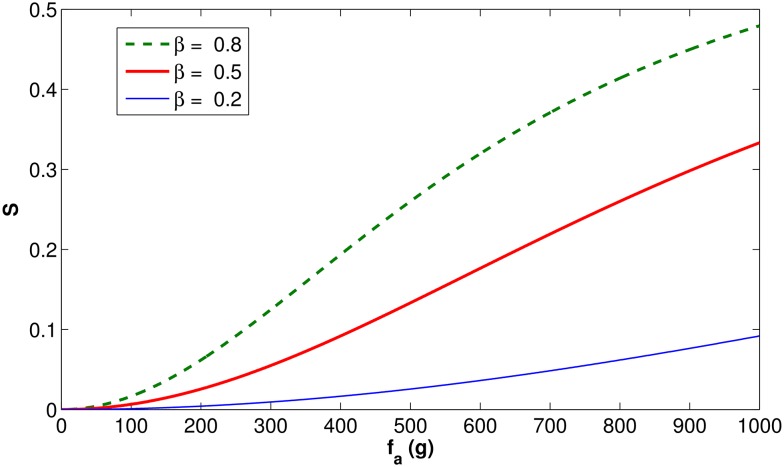
Function *S*(⋅) behavior as a function of *f*_*a*_, for different values of *β* and constant values of *f*, *v*, *d*, *b*, and *H*.

Fig 5 illustrates how parameter *β* affects the survival function *S*(⋅) (defined in Eq (3)) when there is no natural food (*f* = 0 g). The following parameters were adopted: *H* = 10000, *v* = 5000, *d* = 500 g, *b* = 500 g. The figure shows that the higher the value of *β*, the faster *S*(⋅) converges to 1 as more artificial food is added. This means that artificial food of better quality (higher *β*) leads to better survival rate of the queen’s eggs (i.e. an increased percentage reach the adult stage).

### Simulated Scenarios

The scenarios were simulated using MATLAB R2012b. For this purpose, Eqs (1)–(10) were implemented and the parameters specified later in this section were set to run the numerical simulations. The standard Dormand-Prince method [26] was used to solve the differential equations.

#### Model Parameters

As in [16, 17], we consider that the daily rate of egg-laying by the queen is *L* = 2000. The consumption of food is adopted as *γ*_*A*_ = 0.007 g/day (adults) and *γ*_*B*_ = 0.018 g/day (brood). Parameter *ν* is adopted as *ν* = 5000. *α*_*min*_ is 0.25/day, meaning that 4 days (1/*α*_*min*_) is the minimum age at which a hive bee is recruited to become a forager. *α*_*max*_ is considered equal to *α*_*min*_, which implies doubling the rate of recruitment when there is no food available. *σ* is set to 0.75/day, meaning that, when there is no food shortage, foragers will revert to hive bees if more than one third of the total bees are foragers. *ϕ* = 1/9 day^−1^, meaning that 9 days are required for an egg to become a pupa. *τ* = 12 is the number of days required for a pupa to become an adult.

Following [17, 18], the maximum amount of food collected daily by each forager is adopted as *c* = 0.1 g.

A value of *b* = 500 g was proposed in [17] such that the effect of low natural food availability is not perceived if there is at least 1 kg of natural food in the hive. Similarly, we adopt *d* = 500 g.

In these illustrative scenarios, we have set *β* = 0.8, meaning that 1 gram of artificial food is able to replace 0.8 gram of natural food.

#### Scenario 1: Maximum foraging, no artificial food

We start our simulations with no natural or artificial food in the hive, no brood, 16000 hive bees, 8000 foragers, and a daily mortality rate *m* = 0.1. We adopt *μ*(⋅) = 1 to set maximum possible foraging. In other words, there is no natural food shortage in the environment, allowing the forager bees to bring to the colony as much pollen and nectar as they can carry. No artificial food is added to the hive, i.e., *z*(⋅) = 0 g/day. The model was run for 40 days. The results obtained for this set of parameters are the same as those obtained with the model from [17], which indicates consistency between the models when there is maximum foraging (*μ*(⋅) = 1) and no artificial food (*f*_*a*_(*t* = 0) = 0 g, *z*(⋅) = 0 g/day).

#### Scenario 2: Artificial food when there is plenty of natural food

**Fig 6:**
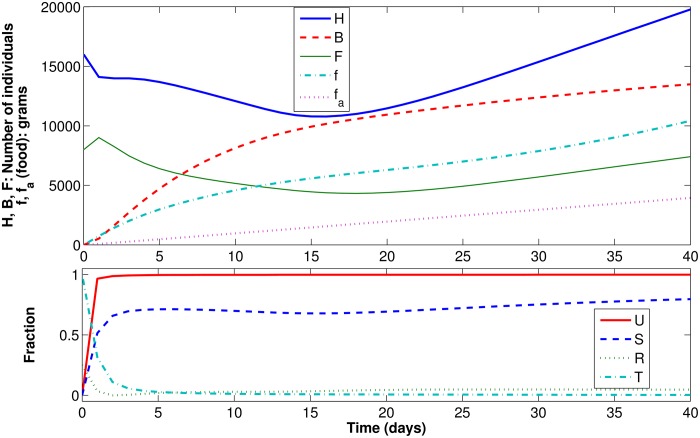
Dynamic results for Scenario 2.

In this second scenario, we keep the conditions of the previous case, but set *z*(⋅) = 100 g/day, meaning that 100 g of artificial food are added to the colony every day. The results are presented in Fig 6. The results show that artificial food is not consumed. The evolution of *B*, *H*, *F*, and *f* is the same that was obtained in the previous case, and *f*_*a*_ grows linearly and reaches 4 kg by the 40th day (that is, all artificial food added by the beekeeper remains in the hive). In fact, the value of *U*(*f*, *f*_*a*_), which is initially 0 (because *f* = *f*_*a*_ = 0 g at day 0), converges very quickly to 1, indicating that all consumed food was of natural origin.

Therefore, our model shows that, when there is plenty of natural food, bees will not feed on the artificial provisions, as expected. These results are consistent with experimental results, in which bees offered artificial diets did not feed from them and chose natural food instead [25].

#### Scenario 3: Shortage of natural food, no artificial food

**Fig 7:**
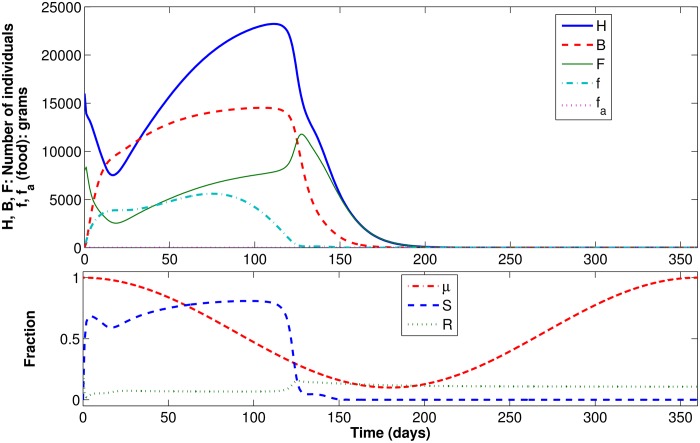
Dynamic results for Scenario 3.

In this third scenario, we maintained the conditions of Scenario 1, but increased the mortality rate to *m* = 0.2 and adopted a sinusoidal function *μ*(⋅), varying from 0.1 to 1, to represent a seasonal shortage of food. The simulation is run for 360 days, starting in mid-summer, when maximum efficiency in collecting food is achieved. Fig 7 presents the results, as well as function *μ*(⋅). It is shown that, around day 116, the survival function *S*(⋅) decreases and the recruitment function *R*(⋅) increases. However, this increase in *R*(⋅) is not enough to compensate for the lack of food in the environment. Food in the colony becomes very scarce, leading to a significant reduction in the number of brood reaching pupation. By day 200, the colony collapses (there are no more hive bees, foragers, or brood).

#### Scenario 4: Shortage of natural food, artificial food added in winter

In this fourth scenario, we repeat the conditions of Scenario 3, but include artificial feeding. We adopt a function *z*(⋅), indicating that during a four-month period, the beekeeper will feed the colony with 400 g of artificial food every seven days. For this purpose, function *z*(⋅) is modeled as a pulse train with a period of 7 days. Each pulse has an amplitude of 400 g/day and width of 1 day, such that the integral of *z*(⋅) over each 7-day window equals 400 g. The four-month period represents the time during which there is shortage of natural food, and might represent, for instance, the last days of autumn, all winter and the first days of spring. This simulation was run for three years.

**Fig 8:**
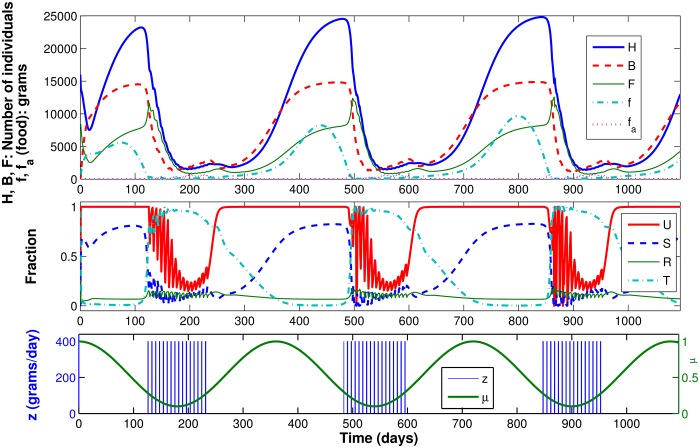
Dynamic results for Scenario 4.

Fig 8 presents the results, as well as functions *z*(⋅) and *μ*(⋅). The figure shows that when there is a shortage of natural food the survival function *S*(⋅) decreases. A few days later, the beekeeper begins artificial feeding, and the *U*(⋅) function decreases from 1, indicating that the bees start to feed not only on natural food, but also on artificial food. This behavior continues when there is little natural food, until *U*(⋅) becomes 1 again at day 278 (indicating that the bees are feeding only on natural food). The number of bees generated in this model shows that artificial feeding is sufficient to keep the colony alive and avoid collapse due to shortage of natural food. The simulation run for a three-year period shows that if artificial feeding is repeated every year, the colony can survive the same harsh conditions that led to colony collapse in Scenario 3, in which there was no artificial feeding.

It is worth noting that Perry *et al.* [20], in spite of adopting a different approach, also have concluded that in-hive feeding rescues vulnerable hives.

### Validation with experimental data

In order to validate the proposed model, simulation results have been compared with the experiments described by Stanger and Laidlaw [27], who studied the effects of different supplemental diets on bees. For this purpose, they analyzed the results of 40 colonies in a five month period, 12/Oct to 16/March, which includes all winter. The colonies were divided in eight groups of five, each group receiving a different formula.

We have considered the first 20 experimental colonies described in [27], that is, colonies submitted to formulas 1 to 4. In each group of five colonies, we have selected the first two as a training set for our model; the other three colonies compose the test set.

In order to reproduce the experiment, the model was run for 155 days with each colony being fed 454 g of artificial food every week.

In [27], the number of bees was measured in number of frames. In order to convert it to number of individuals, we have estimated 2000 adult bees per frame, of which one third were foragers initially.

The total amount of brood was measured in [27] using area. Since it is mentioned in the literature (page 16 of [28]) that 3000 brood cells correspond to 750 *cm*^2^, we have adopted the same ratio, that is, 4 brood cells per *cm*^2^. The areas of unsealed and sealed brood were not described separately in [27]. Since a precise value was not measured, we have assumed that half the brood area described in [27] refers to uncapped brood.

Stanger and Laidlaw [27] reported a sharp decline in the amount of pollen collected from 2 November (day 21). From 30 November (day 49) to 25 January (day 105) there was no pollen being brought into the colonies. From 25 January, the pollen flow increased sharply. Based on this description, we have built the following *μ* function:

$$\begin{array}{c} \mu \left(t\right)=\{\begin{array}{cc} 1, & t<21\\ 0.1, & 21\leq t<49\\ 0, & 49\leq t<105\\ 1, & t\geq 105 \end{array} \end{array}$$ (11)

As in [17], the following model parameters were chosen: *L* = 2000, *γ*_*A*_ = 0.007 g/day, *γ*_*B*_ = 0.018 g/day, *ν* = 5000, *α*_*min*_ = *α*_*max*_ = 0.25 day^−1^, *σ* = 0.75 day^−1^, *τ* = 12 days, *ϕ* = 1/9 day^−1^, *c* = 0.1 g, *m* = 0.06, *b* = 500 g.

The parameter *β* was estimated following experimental data from the training set. To that end, simulations were run varying the value of *β*, initially from 0.10 to 1.00 in steps of 0.05, and then performing a fine tuning in steps of 0.01. The value of *β* that led to a best fitting of the training data was selected.

The estimation of *β* from experimental results is required because the quality of the artificial food needs to be tested in practice, preferably in the field, but a less costly alternative could be to test the supplementary diet feeding the bees in the laboratory.

Since *d* is a parameter associated to palatability, it should be different for each artificial formula. However, for the sake of simplicity, we have chosen to adopt the same value of *d* in all the simulations. As in the previous examples, *d* was set to 500 g. Future works could exploit how the choice of *d* affects the final model outcome and how its value can be estimated from experimental data.

The initial values of *H*, *F* and *B* in each simulation were set considering the initial values described in [27], in which a unique set of initial conditions is described for each colony. Results were evaluated in terms of the final number of adult bees *N* (*N* = *H* + *F*) and brood items *B*.

Table 1 shows the results obtained with the training set, compared with the experimental results from Stanger and Laidlaw [27], as well as the value of *β* estimated for each formula from tuning the model to the training sets. For original experimental results, please refer to Table 1 of [27].

**Table 1:** Comparison between simulated and experimental results (Training Set).

Formula	*β*		Experimental Result [27]	Simulated Result	Difference (%)
1	0.92	*N*	33000	33885	2.7
		*B*	16770	14037	16.3
2	0.65	*N*	29000	28830	0.6
		*B*	12480	13366	7.1
3	0.65	*N*	35000	30492	12.9
		*B*	17221	13607	21.0
4	0.60	*N*	30000	31900	6.3
		*B*	13907	13796	0.8

A single simulation has been run for each formula, intending to represent the average experimental results corresponding to that formula. Each initial value adopted at each simulation, as well as each experimental value of *N* and *B* seen in Table 1, is a mean of the values corresponding to the two first colonies presented in [27].

Table 2 displays the simulated and experimental results obtained with the test set. As in the previous case, a single simulation has been run for each formula, intending to represent the average experimental result.

**Table 2:** Comparison between simulated and experimental results (Test Set).

Formula	*β*		Experimental Result [27]	Simulated Result	Difference (%)
1	0.92	*N*	30000	31327	4.4
		*B*	14189	13718	3.3
2	0.65	*N*	24667	29644	20.2
		*B*	15089	13487	10.6
3	0.65	*N*	30667	30761	0.3
		*B*	14233	13644	4.1
4	0.60	*N*	22000	27681	25.8
		*B*	10642	13188	23.9

The values of *β* presented in Table 2 are the ones previously tuned with the training set. Each initial value adopted at each simulation, as well as each experimental value of *N* and *B* seen in Table 2, is a mean of the values corresponding to the three last colonies presented in [27]. The results in Table 2 show a good match between simulated and experimental results for the test sets.

The highest value of *β* (*β* = 0.92) has been obtained for formula 1, indicating that this is the formula with highest quality. This result is consistent with experimental observations from Stanger and Ladislaw [27], which reported this formula as the best one.

**Fig 9:**
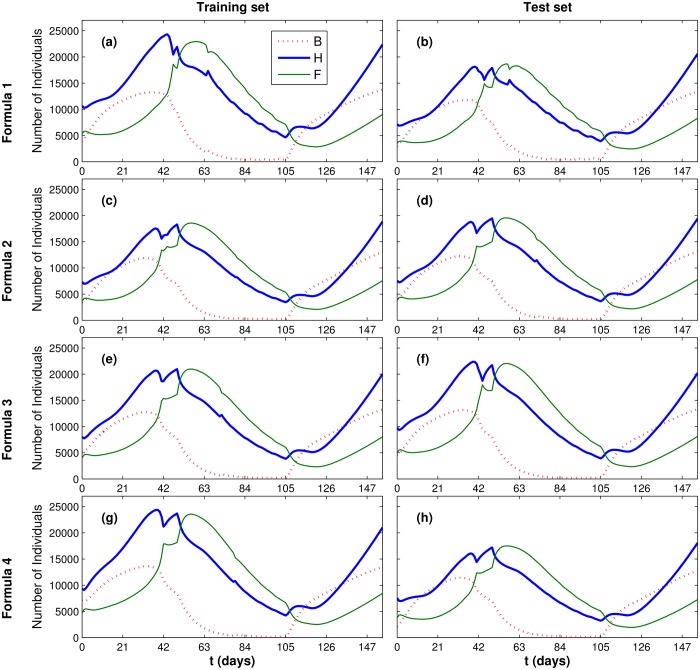
Dynamic results for the simulations representing the experimental scenario.

Fig 9 presents the simulation results for each of the four formulas and for the training and test sets. Time was marked on the x-axis of Fig 9 in steps of 21 days, each one representing a three-week interval.

Fig 9 shows that, at the beginning of each simulation (when there is enough natural food available) the number of brood cells, hive bees and forager bees increases. When natural food starts to become scarce, more hive bees are recruited to become foragers, to compensate for the lack of natural food. Reduction in the number of hive bees *H* and in the availability of natural food causes the number of uncapped brood cells to decrease continually. During the dearth period (days 49 to 105, see Eq (11)), *B* decreases significantly. When there is abundance of pollen again, starting at day 105, the ratio *F*/*H* is reduced and a significant increase in the number of uncapped brood cells *B* is observed.

It is important to highlight that, in this example, the experiments to measure the training and test sets were performed at the same time, that is, under the same exact environmental conditions. A better approach would be to adopt training and test sets measured in different periods of time (for instance, in the winter of different years). This could not be done at this time for lack of experimental data, but it remains as a suggestion for future works.

## Conclusion

This paper has proposed a new model to simulate the influence of artificial feeding on bee colony population dynamics. This model was based on a well-established framework and introduced new elements that allow for analysis of artificial diets, which had not been done before.

A differential equation describing variation in artificial food was included in the formula. The preference of the bees for natural food was modeled using a function *T*(*f*), which includes a parameter *d* associated with the palatability of the artificial diet. A generic function *μ*(⋅) was adopted to account for the lack of natural food due to environmental conditions. A function *z*(⋅) was used to model the approaches adopted by the beekeeper to introduce artificial diet in the colony. A parameter *β* was introduced to quantify the quality of the artificial food.

The proposed model was shown to be consistent with the previous model when there is no artificial food and the foragers gather food with maximum efficiency. The model assumes that, when there is abundance of natural food, the artificial diet is neglected by the bees, as it has been observed in practice. Simulations have shown that this assumption was implemented correctly. Simulations have shown also that, by introducing artificial diets during harsh times (when there is severe shortage of natural food), the beekeeper can avoid the hive collapse that would have occurred if the bees were left to fend for themselves.

Experimental results, involving four different artificial diets, were analyzed to validate the proposed model. The experimental results were divided in training and test sets. The parameter *β* was tuned using the results from the training sets. A good match was found between the results obtained with the model and the results observed experimentally in the test sets.

Future studies could apply the proposed model to other environmental conditions and compare results with new experimental data. Experiments to determine the value of *β* for new diets could be conducted in the laboratory (feeding bees with the artificial diet) and validated in the field. Furthermore, studies could be carried out to assess whether the value of *β* can be estimated based on nutritional parameters of the artificial diet. Finally, the approach proposed here to analyze the effects of artificial food could be integrated into other bee colony population models.
